# Hydrothermal Transformation of Organic Matter in the Case of Domanik Shale Deposits

**DOI:** 10.3390/molecules31081239

**Published:** 2026-04-09

**Authors:** Yaroslav Onishenko, Arash Tajik, Alexey Vakhin, Aleksey Dengaev, Facknwie Kahwir Oscar, Sergey Sitnov, Yulia Duglav, Mustafa Ismaeel, Oybek Mirzaev, Firdavs Aliev

**Affiliations:** 1 Institute of Geology and Petroleum Technologies, Kazan Federal University, 420008 Kazan, Russia; 2 Department of Petroleum Engineering, Gubkin National University of Oil and Gas, Leninskiy Prospect 65, 119991 Moscow, Russia; 3 Department of Chemical Technology, Fergana State Technical University, Fergana 150100, Uzbekistan

**Keywords:** Volga–Ural petroleum province, Domanik shales, kerogen, pyrolysis, maturation, aquathermolysis, hydrothermal treatment

## Abstract

The presence of source rock with a high concentration of kerogen is not a sufficient condition for petroleum formation, as maturation requires specific thermodynamic conditions. In this study, the artificial maturation of organic matter was investigated through hydrothermal treatment simulating the vaporization–condensation zones associated with in situ combustion and steam-assisted recovery processes. The experiments were conducted under an inert nitrogen atmosphere at 250–350 °C to reproduce oxygen-depleted thermal environments where hydrothermal reactions dominate. The results demonstrate that the bitumoid yield increases with temperature, reaching a maximum of 4.44 wt.% at 300 °C, followed by a decline at 350 °C due to secondary cracking. At the same time, gas generation increases significantly, with a more than five-fold rise in total gas yield between 250 and 350 °C. In parallel, the H/C atomic ratio of kerogen decreases from 1.17 in the initial sample to 0.52 at 350 °C, indicating progressive aromatization and advanced catagenetic transformation. These changes are accompanied by the conversion of high-molecular-weight kerogen into resins, asphaltenes, and subsequently lighter hydrocarbons. The study provides experimental evidence for the effectiveness of hydrothermal processes in inducing kerogen transformation under inert conditions, offering insights into the mechanisms governing artificial maturation in unconventional reservoirs.

## 1. Introduction

The carbonate-siliceous Domanik shale deposits of the Volga–Ural Basin are considered a promising alternative source of hydrocarbon resources. However, the potential of such sources can be realized only through the thermal transformation of insoluble organic matter—kerogens—involving both the desorption of hydrocarbons from the surface of kerogen inclusions and the conversion of kerogen itself, yielding synthetic shale oil. The maturation of kerogen represents a gradual transformation within sedimentary strata, controlled by progressive burial and thermal influence over geological timescales. This evolution is primarily governed by the combined effects of temperature, pressure, and duration, and is conventionally divided into three principal stages: diagenesis, catagenesis, and metagenesis, collectively referred to as the ‘oil window’. Such conditions may arise naturally within synclinal zones and in the axial parts of large depressions, but they can also be artificially induced through the application of advanced technologies that significantly accelerate the transformation of kerogens into synthetic oils.

The Domanik formation serves as a source rock sequence, within which the distribution of domanikites (TOC > 5%) and domanikoids (TOC < 5%) exhibits vertical differentiation throughout the section. Notably, the former constitutes the predominant lithotype, accounting for 60% of the total source rock volume. The maximum total organic carbon (TOC) content, reaching up to 12.5%, is observed in the black siliceous-carbonate rocks of the Kamsko-Belskoy depression and the Mukhanovo-Erokhovo trough [1]. The generated petroleum partially migrated into the surrounding reservoir rocks, while the remainder was retained within the Domanik source rock. Therefore, the entirety of the Domanik shales can be regarded as a continuous, non-structural hydrocarbon deposit, where oil and gas are trapped within tight reservoirs of limited porosity and permeability [2]. Another distinguishing characteristic of the Domanik shales is the prevalence of Type II kerogen, which is prone to liquid hydrocarbon generation at early catagenetic stages [3]. Understanding the mechanisms of chemical transformation of organic matter in the Domanik formation under hydrothermal conditions is significant and a relevant task. Globally, current industrial practice in the development of unconventional hydrocarbon resources relies predominantly on thermal stimulation techniques, which are most often implemented in combination with hydraulic fracturing and the drilling of horizontal wells [4]. Building on these approaches, the combined technology relies on controlled heating of the reservoir, thereby establishing conditions favorable for the thermal conversion of kerogen into hydrocarbons. At the same time, it enhances the filtration properties of low-permeable kerogen-rich rocks, ensuring more effective hydrocarbon mobilization [5,6,7]. The technology is based on the injection of oxygen-enriched gas into the reservoir, initiating in situ combustion of hydrocarbons within the formation. The heat generated through this process provides the necessary thermal conditions to enhance and expedite kerogen conversion into synthetic oil. In [8,9], pilot test results on the application of in situ combustion technology implemented by JSC RITEK at the Nazymskoye field in the Bazhenov sweet spots (Priobskaya area) were reported. In situ combustion technology in 2016 was pilot- tested in Chichimene (Colombia) [10]; it was concluded that successful practical application of this method was ensured by the formation depth of −2743 m and a high reservoir temperature of −93 °C.

Kayukova et al. [11] carried out model experiments to investigate the conversion of organic matter extracted from carbonate and siliceous-carbonate rocks. Two types of experimental conditions were examined: (a) hydrothermal treatment of rocks at a temperature of 350 °C under carbon dioxide; and (b) pyrolysis at 350 °C and 600 °C in a hydrogen atmosphere. It was established that increasing the pyrolysis temperature promotes the formation of new free organic radicals within the rock samples. At 350 °C, processes of destruction of high-molecular-weight components and insoluble kerogen occur, leading to a significant increase in the content of free hydrocarbons in the rock and to their more complete extraction. These results highlight the potential of hydrothermal technologies to unlock additional petroleum hydrocarbons from the carbonate-siliceous source rocks of the Domanik Formation [12].

Recent advances in enhanced oil recovery (EOR) technologies emphasize the importance of reservoir stimulation and thermally induced transformation processes for improving hydrocarbon recovery from unconventional resources. Techniques such as hydraulic fracturing, drilling optimization, and flowback analysis have been extensively studied to enhance reservoir performance and evaluate treatment effectiveness. In parallel, hydrothermal conversion approaches provide a complementary pathway by promoting in situ transformation of kerogen into mobile hydrocarbons. Recent studies have also highlighted the growing role of data-driven and machine learning approaches for screening and optimizing EOR strategies [13,14,15]. These developments underline the relevance of investigating hydrothermal processes under controlled laboratory conditions as a means of understanding and improving recovery mechanisms in low-permeability, organic-rich formations.

The aim of this study was to stimulate the vaporization–condensation zones of the in situ combustion process and the steam chamber formed during steam-injection techniques using the rock samples extracted from the Volga–Ural petroleum province. Given the minimal oxygen content within these zones, the experimental series was conducted under an inert atmosphere at temperatures of 250, 300 and 350 °C. For each experiment, both quantitative (material balance) and qualitative (variations in structural-group composition) characterization of the organic matter was performed.

## 2. Results and Discussions

**Figure 1:**
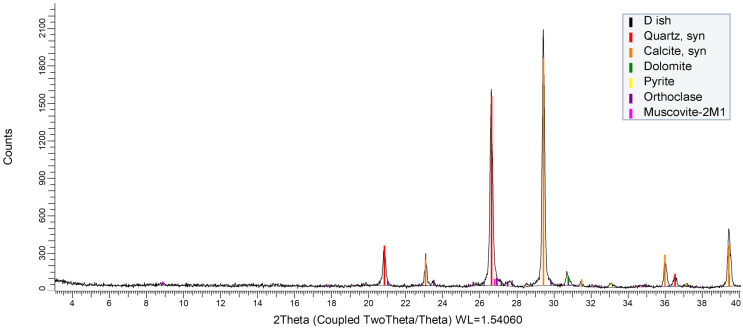
XRD pattern of the initial Domanik shale.

According to X-ray diffraction (XRD) phase analysis conducted using an automatic powder diffractometer, the mineral composition of the original rock was determined and presented in Figure 1. The XRD analysis results indicate that the native rock sample consists of carbonate-siliceous minerals, with an admixture of pyrite, feldspar, and mica. The total organic content in the sample is 11.36 wt.%, of which 2.04 wt.% corresponds to mobile hydrocarbons (bitumoids) and 9.32 wt.% to immobile hydrocarbons (kerogens).

The core mineral composition is predominantly carbonates (calcite 54%, dolomite 2%) and quartz (29%). The contents of potassium mica (muscovite) and potassium feldspar are both 7%. The proportion of pyrite is minor, amounting to 1%.

The results of the SARA analysis of bitumoids, extracted from the core in a Soxhlet apparatus, are presented in Table 1. The high content of high-molecular-weight components (resins and asphaltenes) in the bitumoid of Domanic shales (67.36 wt.%) is corroborated by a number of studies [16,17].

The result of elemental analysis of the isolated kerogen is presented in Table 2. The formation of the gas phase results from the breaking of C-C and C-heteroatom bonds during the autoclave experiments. Within the studied temperature range, in addition to hydrocarbon gases, the formation of CO, CO_2_, and H_2_S was specific.

The gas chromatography results are presented in Table 3. The total gas volume increases with temperature, rising from 0.402 g at 250 °C to 2.187 g at 350 °C. The formation of hydrogen sulfide at temperatures above 300 °C indicates the destruction of sulfide and disulfide bonds in kerogen and heteroatomic compounds present in the bitumen, as well as the thermal decomposition of thiols [18].

**Figure 2:**
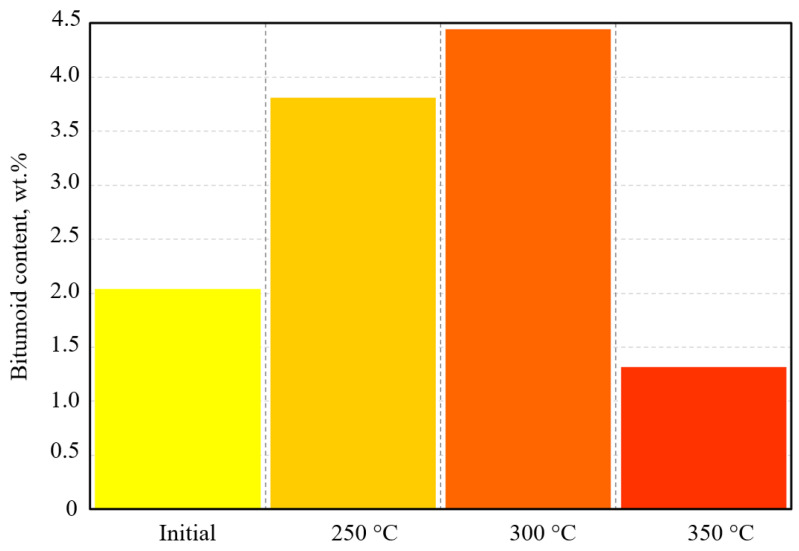
The content of bitumoids in initial oil sample and after upgrading at different temperatures.

With increasing temperature, the content of hydrocarbon gases ranging from nC_1_ to nC_6_ increases markedly, which is attributed to the cleavage of alkyl peripheral groups within the kerogen structure and the resin and asphaltene fractions of the bitumen. Following the autoclave experiments, the mass content of bitumoid and its group composition (saturated hydrocarbons, aromatic hydrocarbons, resins, and asphaltenes) were altered (Figure 2; Table 4 and Table 5).

The mass of initial bitumoid was 2.04 wt.%; 41.75 wt.% and 28.22 wt.% from this bitumoid accordingly corresponded to resins and asphaltenes. The amount of saturates in it was only 6.43 wt.%.

As the experimental temperature increased, the content of bitumoid rose accordingly. At 250 °C, it amounted to 3.81 wt.% and reached its maximum value of 4.44 wt.% at 300 °C, and then sharply decreased to 1.32 wt.% at 350 °C.

The mass of residual kerogen after autoclave experiments at 250 and 300 °C decreased as within this temperature range, high-molecular-weight heteroatomic (N, S, and O) compounds were primarily released from the kerogen. These compounds were represented in the bitumoid composition by resins and asphaltenes, as confirmed by SARA analysis of the bitumoids.

The asphaltene content increased from 28.2 wt.% to 41.6 wt.% after the experiment at 250 °C, while the proportion of low-molecular-weight hydrocarbons decreased: the saturated hydrocarbon fraction dropped from 6.4% to 6.2%, and the aromatic hydrocarbon fraction decreased from 23.6% to 19.6%. This is attributed to the introduction of high-molecular-weight kerogen degradation products into the system. As the experimental temperature increased to 350 °C, saturates content rose from 6.4 wt.% to 17.8 wt.%, while the resins and asphaltenes decreased by 13% and 10%, respectively. These changes in the composition of bitumoids are associated with the destruction of C-C bonds in the residual kerogen and the cracking of newly formed resins and asphaltenes, leading to the generation of low-molecular-weight hydrocarbons [19].

**Figure 3:**
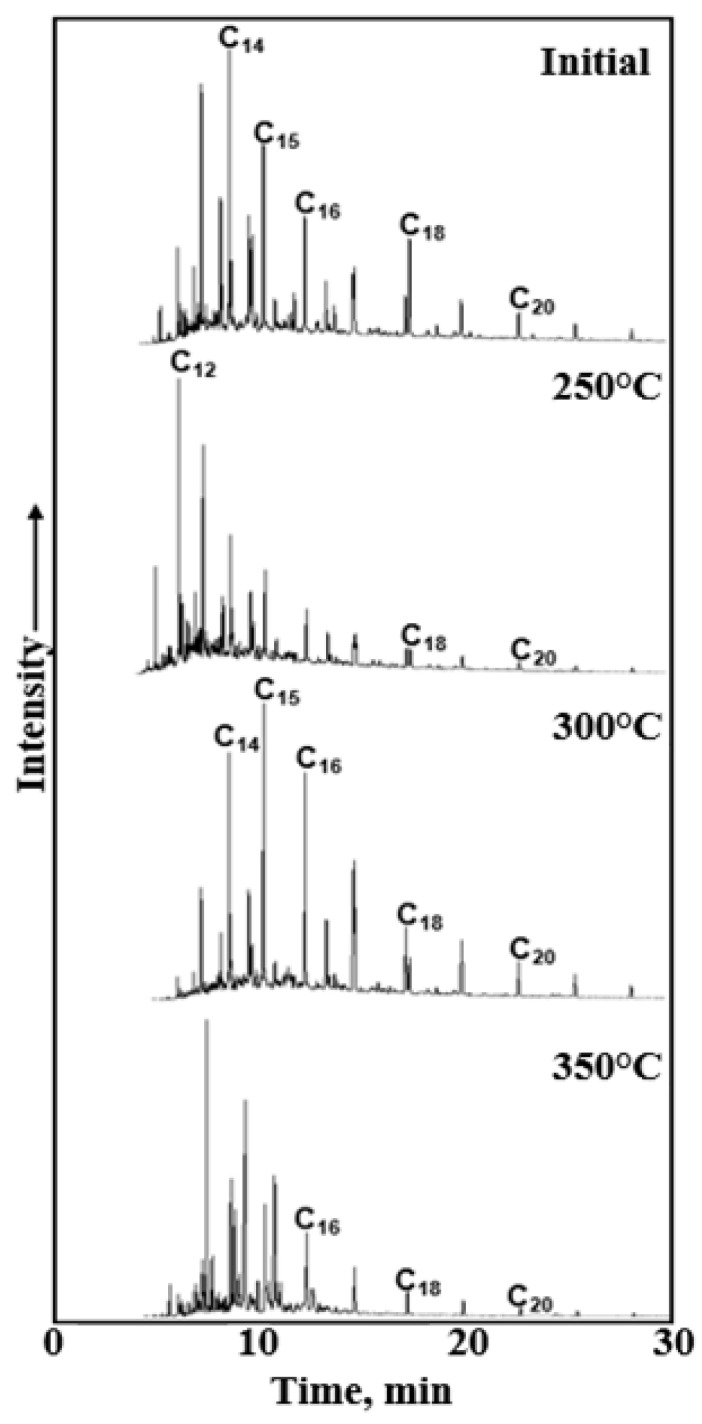
GC-MS spectra of the saturated hydrocarbons of the initial bitumoid and after hydrothermal treatment (TIC).

Based on GC-MS results, the saturated composition is dominated by n-alkanes with even carbon numbers (Figure 3). The ratio of mid-molecular-weight (C_10_–C_19_) to high-molecular-weight n-alkanes (C_20_–C_30_) in the initial crude oil sample was 6.3 (Table 6). This ratio increases non-uniformly with the rise in upgrading temperature. At a temperature of 250 °C, the ratio increased to 11.06, which was likely associated with the desorption of hydrocarbons predominantly of medium molecular weight. In the temperature range of 300–350 °C, a decrease in the ratio down to 7.50 was observed, resulting from the addition of high-molecular-weight hydrocarbons due to the cracking of kerogen and newly formed resins and asphaltenes.

For the initial bitumoid sample, the Pr/Ph ratio was 0.53 (Table 6), which is consistent with the formation of kerogen in the Domanik deposits under reducing anoxic conditions typical of a marine environment [20].

Furthermore, the Pr/Ph ratio was used to assess the maturity level of organic matter. As the maturity degree of organic matter increased, the Pr/Ph ratio rose from 0.53 (initial sample) to 1.25 after the experiment at 300 °C. However, starting at a temperature of 350 °C, an inversion in the content of isoprenoids occurred, and consequently, this ratio decreased. Bazhenova and Shapiro [21] proposed a calculation of the organic matter maturity coefficient using the following Formula (1):

(1) $$K_{i}=\frac{PrPr+Ph}{H-C_{17}+H-C_{18}}$$

With increasing catagenetic maturity of the kerogen, the coefficient value decreases. For the initial sample, *K_i_* = 1.60; as the temperature rose to 350 °C, it decreased to 0.12, which overall confirms the maturity assessment based on the P_r_/P_h_ ratio (Table 6). The P_r_/nC_17_ and P_h_/nC_18_ ratios, which were used to determine the conditions of petroleum formation, decreased with increasing kerogen maturity (up to a temperature of 350 °C), and this trend was consistent with the findings reported by Alexander et al. [22].

In addition to the geochemical characteristics determined by the composition of n-alkanes and isoprenoids, Table 6 also presents biomarker hydrocarbon ratios reflecting changes in the maturation degree.

At 250 °C, the initial increase in Pr/Ph from 0.53 to 1.45 reflected the preferential release of isoprenoids (pristane and phytane) from the kerogen matrix through cleavage of relatively weak C–S, C–O, and C–N bonds, as well as desorption of entrapped hydrocarbons. This stage was dominated by the physical liberation and mild thermal release of isoprenoids without extensive cracking of aliphatic chains [23,24].

**Figure 4:**
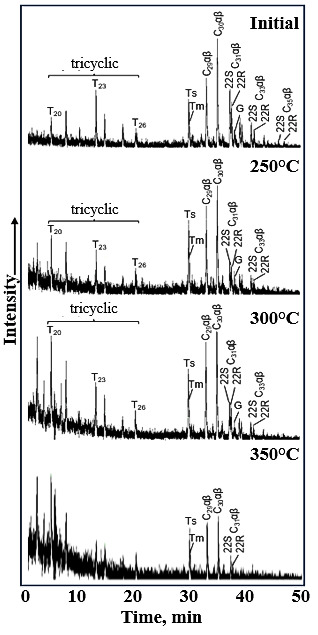
GC-MS spectra of terpane hydrocarbons of the initial bitumoid and after hydrothermal treatment (*m*/*z* = 191).

At 300 °C, the Pr/Ph ratio remained elevated (1.25), consistent with ongoing kerogen decomposition. However, the reduction in the Σisoprenoids/Σn-alkanes ratio (Table 6) indicated that the rate of n-alkane generation begins to outpace that of isoprenoid release. This is attributed to the onset of homolytic cleavage of C–C bonds in aliphatic side chains and naphthenic structures within kerogen and newly formed asphaltenes/resins. At 350 °C, the Pr/Ph ratio declined to 0.82. This inversion is explained by more severe thermal stress: pristane and phytane themselves undergo secondary cracking, converting into lower-molecular-weight hydrocarbons, thereby reducing their absolute abundance. The increase in saturates content from 15.4 wt.% (300 °C) to 17.8 wt.% (350 °C) (Table 5), coupled with a sharp rise in the Σisoprenoids/Σn-alkanes ratio (from 3.13 to 13.90), actually indicates that n-alkane concentration increased significantly, diluting the relative abundance of isoprenoids. This was supported by the decrease in the Pr/n C_17_ and Ph/n C_18_ ratios (Table 6). Elemental analysis (Table 7) shows a drop in H/C atomic ratio from 0.72 (300 °C) to 0.52 (350 °C). This indicates advanced aromatization, which reduced the availability of aliphatic precursors for isoprenoid preservation. Changes in terpane parameters (e.g., Ts/Tm, βα C_30_) and the dominance of low-molecular-weight terpanes (C_19_, C_21_) over C_23_ in GC MS (Figure 4) confirmed that thermal stress reached a level where classical biomarker ratios were altered due to both cracking and isomerization.

The composition of tricyclic terpanes in the bitumen was redistributed after hydrothermal treatment (Figure 4). The changes in the aforementioned polycyclic hydrocarbons are associated with alterations in chiral centers during artificial maturation and the subsequent increase in concentrations of thermodynamically stable diastereomers. In the initial sample, triterpanes of composition C_23_ predominated; however, after hydrothermal experiments, terpanes of composition C_19_ and C_21_ became dominant.

**Figure 5:**
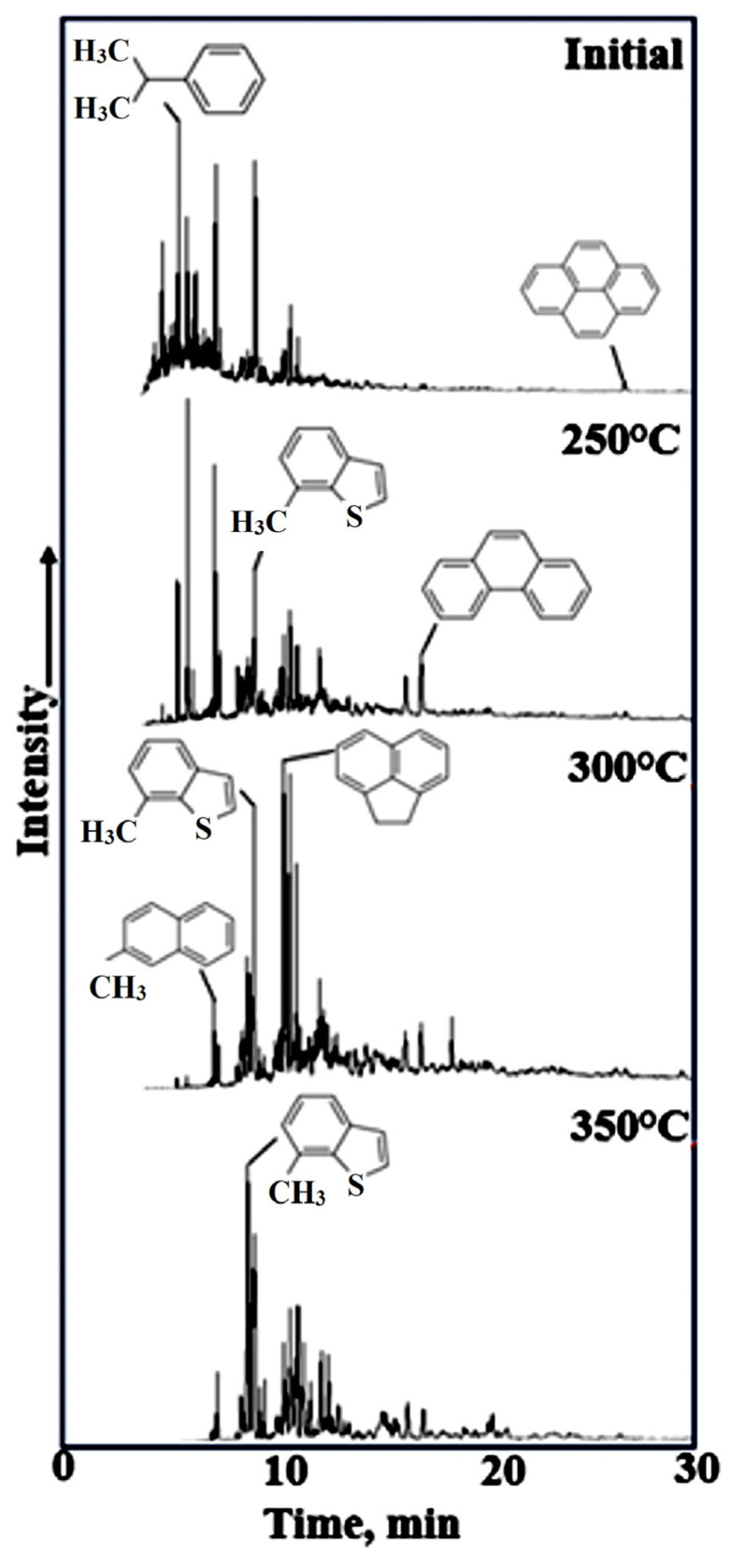
GC-MS spectra of the aromatic hydrocarbons of the initial bitumoid and after hydrothermal treatment (TIC).

Cumene homologs predominated in the aromatic fraction of the initial oil sample; however, following autoclave experiments, homologs of polyaromatic compounds, such as naphthalene, acenaphthene, phenanthrene, and pyrene, became predominant (Figure 5).

**Figure 6:**
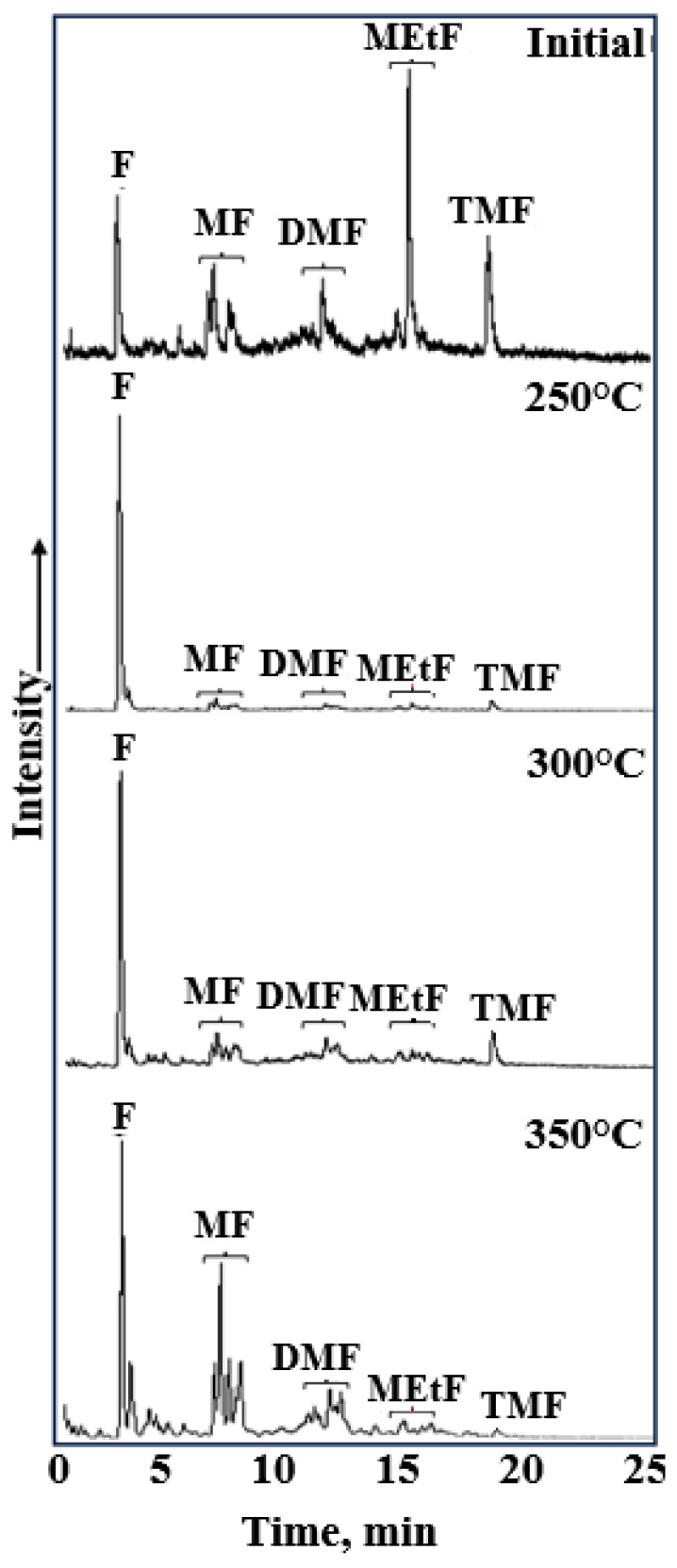
GC-MS spectra of phenantrenes (F), methyl—(MF), dimethyl—(DMF), and trimethyl (TMF) homologs of initial bitumoid and after hydrothermal treatment (*m*/*z* = 178 + 192 + 206 + 220).

In the initial bitumen sample, the ratio of phenanthrene (P) to methylphenanthrenes (MP) to dimethylphenanthrenes (DMP) was characterized by a predominance of methyl-substituted derivatives (Figure 6). A similar dominance pattern was observed at 350 °C. However, following autoclaving in the 250–350 °C range, a sharp shift in the distribution occurred, favoring monocyclic arenes.

GC-MS data reveal that hydrothermal treatment leads to a relative increase in low-molecular-weight components within the saturated and aromatic fractions. This compositional shift enhances oil quality and, consequently, reduces the costs associated with its production and transportation.

According to the literature, the original kerogen is identified as Type II, characterized by an H/C atomic ratio of 1.17 and a Hydrogen Index (HI) of 330.41 mg HC/g TOC. Vandenbroucke and Largeau implied that such a kerogen type originates from microbial organic matter deposited in a relatively deep-water basin [25]. In contrast to Type I kerogen, the macromolecular structure of type II kerogen is characterized by a dominance of polyaromatic nuclei.

Elemental analysis (Table 7) demonstrates a systematic decrease in the H/C_org_ atomic ratio with rising temperature—from 1.17 in the initial sample to 0.72 at 300 °C and 0.52 at 350—clearly indicating progressive catagenetic transformation of the organic matter. This trend reflects the increasing aromatization of the kerogen structure, attributed to the cleavage of alkyl substituents and the associated disproportionation of n-alkanes during thermal decomposition [26].

**Figure 7:**
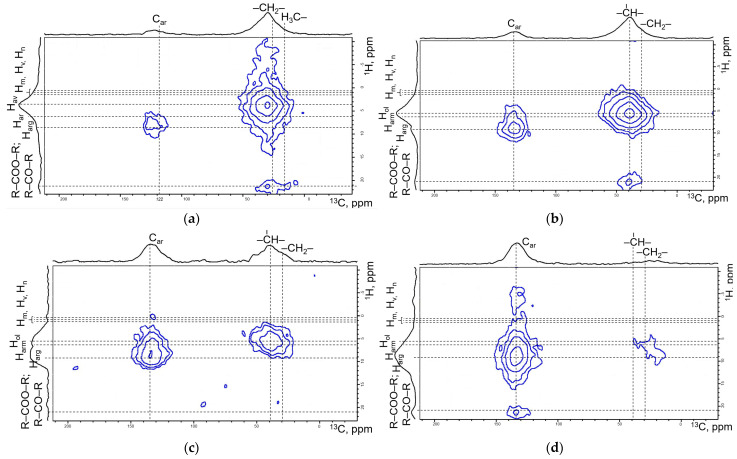
^13^C—NMR spectroscopy of (**a**)—initial kerogen; (**b**)—kerogen after 250 °C; (**c**)—kerogen after 300 °C; (**d**)—kerogen after 350 °C.

T_max_ increased linearly with heating temperature, reaching a maximum of 433 °C at a heating temperature of 350 °C, which indicates a change in the thermal maturity of the kerogen [27]. The content of free hydrocarbons (S_1_) in the initial sample was 1.98 mg HC/g rock. This parameter increased with rising heating temperature up to 300 °C, reaching 7.22 mg HC/g rock. However, at a temperature of 350 °C, the free hydrocarbon content decreased to 5.31 mg HC/g rock. The S1 values correlated with the yield of bitumen following thermal treatment (Table 7). The kerogen generation potential (S_2_) systematically decreased with increasing heating temperature due to the cleavage of C-S, C-O, C-N, S-S, and C_aromatic_-C_aliphatic_ bonds. The initial kerogen possessed a relatively high S2 value of 224.41 mg HC/g rock. At 300 °C, this value decreased to 79.84 mg HC/g rock, and at 350 °C, it was 11.63 mg HC/g rock. As a result of the thermal decomposition of kerogen, the intensity of aliphatic groups diminished, and polyaromatic structures became predominant. The primary indicator of the petroleum potential of a source rock was the maturity of its kerogen, along with changes in its composition and chemical structure resulting from maturation processes. Correlation between the chemical shifts obtained from ^13^C and ^1^H NMR spectroscopy allowed for the identification of the most significant structural changes, offering advantages over 1D ^13^C or ^1^H-NMR spectra alone. Figure 7 presents two-dimensional 1H-13C NMR spectra of kerogen isolated from Domanik rock, both in its initial state and after hydrothermal treatment.

In the NMR spectra (Figure 7), the regions corresponding to aliphatic (10–40 ppm) and aromatic (100–150 ppm) carbon can be distinguished [28,29].

In the initial kerogen, the maximum signal intensity in the aliphatic carbon region occurred at 27.5–37.0 ppm, corresponding to -CH_2_- groups in alkyl compounds. A signal in the range of 18.0–20.5 ppm is attributed to an α-CH_3_ group shielded by one aromatic ring. In the aromatic carbon region (118.0–129.0 ppm), the signal intensity of protonated carbon atoms was minimal. According to ^1^H-NMR spectroscopy data, the maximum signal intensity in the initial kerogen sample occurred in the range of 2.0–4.0 ppm, corresponding to hydrogen protons bonded to aliphatic carbon [30].

The NMR spectra exhibit a progressive decrease in signal intensity in the aliphatic carbon region after hydrothermal upgrading (Figure 7), with minimal intensity observed in this region at 350 °C. As the experimental temperature increased, chemical shifts were recorded for hydrogen protons of alkyl and methyl groups in the α-position relative to aromatic nuclei (from 1.0–4.0 ppm to 6.0–9.3 ppm, characteristic of protons in mono- and polyaromatic structures). A similar chemical shift was observed for carbon, transitioning from alkyl radicals to carbon within aromatic structures [31].

It should be noted that the experimental approach employed in this study was designed to isolate the thermochemical aspects of hydrothermal transformation under controlled conditions. While fluid migration processes characteristic of natural reservoirs were not explicitly reproduced in a closed autoclave system, this setup enabled a focused investigation of temperature-driven reactions. In addition, the experiments were conducted at a fixed reaction time (24 h) to ensure sufficient product formation; however, further time-resolved studies would be valuable for capturing the kinetics of kerogen transformation and refining the interpretation of reaction pathways.

## 3. Materials and Methods

### 3.1. Main Properties of Domanic Oil Shale

A sample of Domanik rock enriched in kerogen from the Pervomayskoe area (Republic of Tatarstan), belonging to the Frasnian–Famennian carbonate complex, was selected as the object of study. The sampling interval was 1597.0–1601.2 m, with the sample taken at a depth of 0.65 m. According to the classification of kerogens, the Domanik oil shale corresponds to type I/II, with the total organic content ranging between 0.72 and 13.31% [32]. The total porosity of shale rocks varies between 2.4% and 3.3%. The experimentally isolated bitumen content in the original sample was 2.04%, and the kerogen content is 9.32% per 100 g of shale rock, which fits the upper range reported in the literature.

### 3.2. Experimental Set-Up

**Figure 8:**
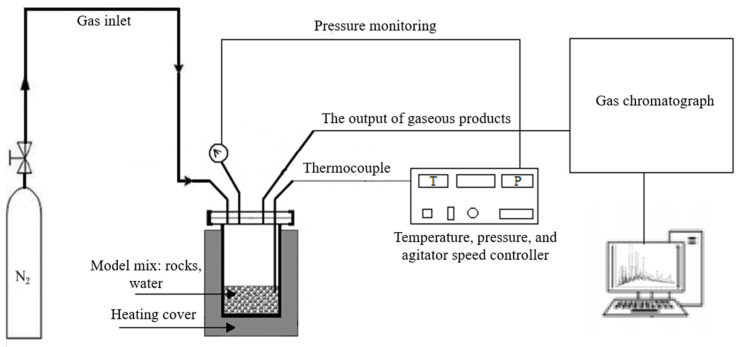
Schematic illustration of experimental set-up.

The hydrothermal transformation of disintegrated kerogen-bearing shale was conducted in a high-pressure and high-temperature reactor equipped with a mechanical stirrer (Figure 8) manufactured by Parr Instruments, Moline, IL, USA. The experiments were performed isothermally at temperatures ranging from 250 to 350 °C, under a constant pressure of 9 MPa for a duration of 24 h. The heating rate was controlled at 5 °C/min until the target temperature was reached. The water-to-rock mass ratio was maintained at 1:10, simulating subsurface conditions characteristic of steam injection processes. Prior to heating, the system was purged with nitrogen gas for 20 min to ensure an inert atmosphere, followed by pressurization and a 15 min leak test to verify system integrity. No mechanical stirring was applied during the experiments. Under the batch reactor conditions, natural convection and thermal diffusion were considered sufficient for the intended water-rock interactions. The HP-HT reactor is coupled with a gas chromatography Crystal 5000.2 (Chromatec, Yoshkar-Ola, Russia) equipped with a thermal conductivity detector and computer-assisted data acquisition to analyze the composition of the evolved gases after hydrothermal treatment and to evaluate the gas yield.

Kerogen isolation was achieved through sequential treatment of the crushed, de-bituminized rock with hydrochloric and hydrofluoric acid solutions. This approach is considered the only reliable method for determining the quantitative kerogen content. Residual bitumen was removed by re-extracting the isolated kerogen with chloroform.

### 3.3. Analytical Methods

X-ray diffraction (XRD) analysis. The phase analysis of the shale rock was performed using a Shimadzu XRD-7000S (Shimadzu, Kyoto, Japan) powder diffractometer with CuKα radiation (λ = 1.54060 nm), employing a nickel monochromator on the diffracted beam, a step size of 0.0008 Å, and an exposure time of 3 s per point. Additional measurements were obtained using a D2 Phaser (Bruker, Berlin, Germany) with identical radiation parameters. Processing of the diffraction spectra and identification of the crystalline phases present were performed using the proprietary interactive computer system EVA (version 4.0), which is designed for the analysis of sedimentary rocks and soils and incorporates specialized ICDD-2010 databases.

SARA analysis. The group composition of the synthetic oil produced through hydrothermal conversion was determined in accordance with the Russian standard GOST 32269-2013, which is analogous to ASTM D4124-09 [33].

Firstly, asphaltenes were precipitated using the cold gold method. For this procedure, each oil sample was mixed with n-hexane in a ratio of 1:40 (oil:solvent). The mixture was stirred for one hour and then allowed to stand for 16 h. Subsequently, the mixture was filtered through paper filters (grade “blue ribbon”). The asphaltenes, which had precipitated overnight, were retained on the filter, while the filtrate containing the maltenes dissolved in n-hexane was collected in a round-bottom flask. To recover any residual maltenes remaining on the filter, hot extraction with n-hexane was performed using a Soxhlet apparatus. The extraction was continued until the refluxing solvent became completely transparent. The n-hexane was then distilled off from the collected extract using a rotary evaporator. Following this, asphaltenes were extracted from the same filter with toluene in the Soxhlet apparatus. Chromatographic separation of the maltenes was carried out using a glass column (20 × 500 mm) dry-packed with neutral alumina, which had been pre-activated by calcination at 450 °C for 3 h. The column was secured vertically, and approximately 50 mL of hexane was introduced to pre-wet the adsorbent. The maltene fraction, redissolved in a minimum amount of hexane, was then transferred onto the column. Three solvents of differing polarity, each with a volume of 200 mL, were sequentially applied to elute the respective fractions: n-hexane for saturated hydrocarbons, toluene for aromatic hydrocarbons, and a toluene–methanol mixture (3:1, *v*/*v*) for resin components. The solvents were subsequently removed from the isolated fractions using a rotary vacuum evaporator to avoid overheating and potential alteration of hydrocarbon structures at elevated temperatures.

Gas Chromatography (GC) analysis. The qualitative and quantitative composition of the gases was determined by a GC Chromatek-Crystall 5000.2 equipped with a flame ionization detector (FID) and thermal conductivity detectors (TCDs) made in Russia. A gas sample was extracted from the autoclave through a gas outlet and transferred to the chromatograph via a dedicated gas line with a pressure reducer. Chromatographic separation was performed using a packed Hayesep Q column (1.5 m × 2 mm, 80/100 mesh), which resolved hydrogen sulfide and carbon dioxide for subsequent detection by TCD-1. Hydrogen, oxygen, and nitrogen were separated on a packed NaX column (2 m × 3 mm, 60/80 mesh) and detected by TCD-2. Hydrocarbon gases were separated on a capillary CR-1 PONA column (100 m × 0.25 mm × 0.5 µm) and detected by the FID. The analysis employed the following temperature program: an initial hold at 35 °C for 13 min, followed by a ramp at 10 °C/min to 45 °C (hold for 15 min), then a ramp at 1 °C/min to 60 °C (hold for 15 min), and finally a ramp at 2 °C/min to 240 °C (hold for 41 min). Helium was used as the carrier gas. The inlet pressure at the capillary column was 323 kPa, while the flow rate through the packed columns was maintained at 15 mL/min. Data acquisition and processing were carried out using Chromatek Analytic 3.1 software (Chromatek, Yoshkar-Ola, Russia). Gas samples were collected directly from the reactor through the gas outlet using a pressure-controlled sampling line. Prior to analysis, the gas chromatograph was calibrated using certified standard gas mixtures containing known concentrations of hydrocarbons (C_1_–C_6_), CO_2_, and H_2_S. Quantitative analysis was performed based on calibration curves obtained for each component. The reproducibility of the measurements was ensured by repeated injections, with deviations within acceptable analytical limits.

Gas chromatography–mass spectroscopy (GC-MS). The structure of synthetic oil constituents after SARA analysis was investigated using a gas chromatograph–mass spectrometric (GC-MS) system comprising a Chromatec-Crystal 5000 gas chromatograph (Chromatec, Yoshkar-Ola, Russia) coupled with an ISQ mass-selective detector.

Sample injection (0.2 µL) was performed using a specialized DAZH-2M (3D) automatic liquid autosampler made in Russia. The liquid sample was introduced directly into the chromatograph’s injector port. Upon vaporization in the heated inlet, the analytes were transported into the chromatographic column by an inert carrier gas (helium), which does not compromise the accuracy of the results. Separation was achieved on a 30 m × 0.25 mm capillary column (Chromatec) with a CR-5ms stationary phase. The chromatography was conducted using the following temperature program: from 100 °C to 150 °C at a rate of 12.5 °C/min, followed by a ramp to 310 °C at 3 °C/min. The carrier gas (helium) flow rate was maintained at 1 mL/min. Following separation, the components entered the mass selective detector (MSD). Data acquisition and processing were performed using Chromatec Analytic 3.1 software (Chromatec).

Isolation of soluble organic matter and kerogen from shales. Prior to the extraction of soluble organic matter, both the initial rock sample and the post-autoclave samples were separated from water and placed in a drying oven at 60 °C to remove residual moisture. Subsequently, oil was extracted from the dried rock using a solvent mixture of chloroform, benzene, and isopropyl alcohol in a 1:1:1 ratio. The extraction of soluble organic matter from the pulverized shale was continued until the circulating solvent became transparent. A Heidolph rotary evaporator was used for solvent recovery. The resulting extract was weighed with a measurement precision of ±0.01 g to establish the material balance. It should be noted that the amount of coke formed during the experiment was not quantified in this study due to the difficulty of separating it from the spent shale from the isolated kerogen; consequently, its mass is included in the mass of the residual rock in the mass of the isolated kerogen.

**Figure 9:**
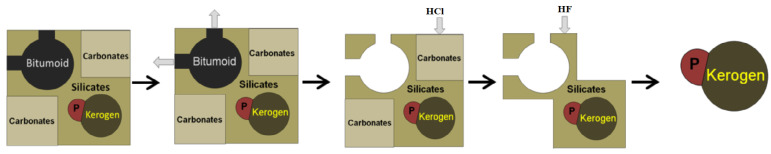
Kerogen isolation as per [34].

Kerogen isolation (Figure 9) was achieved through the sequential removal of debitumenized rock matrices with hydrochloric acid (carbonates) and hydrofluoric acid (silicates) solutions as per [34]. However, after acid treatment, the mineral residue became enriched in quartz and pyrite particles due to their higher inertness. This observation was also reported in [35]. In order to eliminate quartz upon repeated treatment with hydrofluoric acid, the procedure was modified from that of Surikov et al. [36] with the addition of 5 mL of concentrated sulfuric acid to prevent the precipitation of insoluble fluorides.

In order to remove the bulk of the pyrite, chromium (II) chloride was synthesized under laboratory conditions. The pyrite removal reaction proceeded with the release of hydrogen sulfide. This approach is considered the only reliable method for determining the quantitative kerogen content. Residual bitumen was removed by re-extracting the isolated kerogen with chloroform.

Nuclear magnetic resonance spectroscopy (NMR). The structural transformations of kerogen were investigated using solid-state ^13^C nuclear magnetic resonance spectroscopy with an AVANCE 400 III spectrometer (Bruker, Berlin, Germany).

The spectrometer was operated at a proton frequency of 400 MHz. Liquid-state analyses were performed in 5 mm tubes, while both 5 mm and 10 mm tubes were available for 1H studies. For solid-state MAS experiments, samples were packed into 4 mm zirconia rotors and spun at rates up to 18 kHz, covering a resonance frequency range of 40–162 MHz for the observed nuclei. Data were acquired using standard Bruker pulse sequences. Sensitivity in solid-state experiments was enhanced through cross-polarization (CP/MAS) from protons to carbon. Two-dimensional heteronuclear ^1^H–^13^C correlation spectra (HETCOR) were obtained under frequency-switched Lee-Goldburg (FSLG) homonuclear decoupling at 110 kHz.

Elemental analysis. Elemental composition (CHN-O) was determined on a CHN-3 elemental analyzer (PerkinElmer, Shelton, CT, USA) at 1000 °C, while oxygen content was estimated. Sulfur content was additionally verified using a Spectroscan analyzer (SPECTROSCAN, St. Petersburg, Russia).

Pyrolytic analysis. Pyrolytic analysis of the native and thermocatalytically transformed kerogen was performed using a Frontier Lab EGA/PY-3030D pyrolytic system (Frontier Laboratories LTD, Koriyama, Japan).

In this paper, the AI product DeepSeek-V3 by Hangzhou DeepSeek Artificial Intelligence Co., Ltd. (Hangzhou, China) was used for improving the scientific writing style and for superficial text editing.

## 4. Conclusions

Hydrothermal pyrolysis conditions corresponding to the vaporization–condensation zone were simulated in a high-pressure–high-temperature reactor using a Domanik shale sample from the Volga–Ural petroleum province to investigate the transformation of kerogen-bearing source rocks under artificial maturation. Petroleum extracts (bitumoids) were obtained after hydrothermal treatment at 250, 300, and 350 °C, and the corresponding kerogen samples were isolated. The yield of bitumen increased with heating temperature up to 300 °C, reaching a maximum value, and subsequently decreased at 350 °C due to secondary cracking processes leading to increased gas generation and depletion of liquid hydrocarbons. At the same time, the mass of kerogen decreased as a result of its transformation into resins, asphaltenes, and lighter hydrocarbon fractions. A more than five-fold increase in total gas yield was observed between 250 °C and 350 °C, reflecting the progressive cleavage of C–C and C–heteroatom bonds. The presence of CO, CO_2_, and H_2_S in the gas phase confirmed the decomposition of sulfur- and oxygen-containing compounds and structural alteration of the organic matter. Geochemical indicators, including the decrease in the maturity coefficient (Ki) from 1.60 to 0.12 and changes in biomarker ratios, confirmed the progressive catagenetic transformation of kerogen. Furthermore, combined ^1^H–^13^C NMR and pyrolytic GC-MS data indicated a significant increase in aromaticity, reflecting advanced thermal evolution of the organic matter.

## Figures and Tables

**Table 1:** Group composition of bitumoids extracted from original core sample.

Sample	Content, wt.%			
	Saturates	Aromatics	Resins	Asphaltenes
Pervomayskoe	6.43	23.6	41.75	28.22

**Table 2:** Elemental composition of native kerogen.

Sample	Content, wt.%			
	C	H	N	O
Pervomayskoe	67.92	6.64	5.15	5.09

**Table 3:** Composition of the evolved gases after hydrothermal treatment.

Temperature, °C	Content, wt.%									
	CH_4_	C_2_H_6_	C_3_H_8_	C_4_H_10_	C_5_H_12_	C_6_H_14_	CO	CO_2_	H_2_S	Other Gases
250	0.0145	0.0174	0.0157	0.0081	0.0034	0.0013	-	0.166	-	0.1936
300	0.0894	0.0973	0.0744	0.0338	0.0134	0.0053	-	0.294	0.005	0.2914
350	0.3628	0.3173	0.2437	0.0944	0.0284	0.0074	0.0028	0.826	0.052	0.2522

**Table 4:** The content of organic matter in initial rock sample and after hydrothermal treatment.

Temperature, °C	Bitumoid Yield, wt.%	Kerogen Content, wt.%	Gas Yield, wt.%
Initial	2.04	9.32	-
250	3.81	7.50	0.42
300	4.44	4.70	0.90
350	1.32	6.54	2.19

**Table 5:** SARA fractions of bitumoids.

Sample	Content, wt.%			
	Saturates	Aromatics	Resins	Asphaltenes
Initial oil	6.4	23.6	41.8	28.2
250 °C	6.2	19.6	32.6	41.6
300 °C	15.4	22.6	29.5	32.5
350 °C	17.8	35.8	28.8	17.6

**Table 6:** Geochemical parameters of bitumoids.

	Temperature of the Upgrading Process, °C			
	Initial sample	250	300	350
n-alkanes and isoprenoids (*m*/*z* = 57)				
Pr/Ph	0.53	1.45	1.25	0.82
Pr/h-C_17_	1.04	1.23	0.39	0.08
Ph/h-C_18_	2.23	1.06	0.56	0.17
K_i_	1.60	1.15	0.46	0.12
C_10_–C_19_/ C_20_–C_30_	6.30	11.06	7.50	9.74
Σisoprenoids/ Σn-alkanes	1.78	2.09	3.13	13.90
Terpanes (*m*/*z* = 191)				
Ts/Tm	4.54	11.41	9.92	-
βα, % C_30_	8.87	9.22	9.62	13.13
22S/(22S + 22R) C_31_	0.57	0.48	0.57	0.56

**Table 7:** Elemental composition and key parameters of initial kerogen sample and after hydrothermal treatment.

Sample	Elemental Composition, %					Key Parameters					
	C_org_	H	N	O	H/C_org_	T_max_, °C	S_1_	S_2_	HI	IP	PP
Initial	67.92	6.64	5.15	5.09	1.17	419	1.98	224.41	330.41	0.01	226.39
250 °C	67.43	5.74	5.58	6.64	1.02	421	2.69	157.52	233.60	0.02	160.20
300 °C	77.14	4.62	6.36	7.48	0.72	427	7.22	79.84	103.49	0.08	87.05
350 °C	79.93	3.48	6.23	6.44	0.52	433	5.31	11.63	14.55	0.31	16.94

## Data Availability

Dataset available on request from the authors.

## Abbreviations

The following abbreviations are used in this manuscript:

TOC	Total organic carbon content
XRD	X-ray diffraction
SARA	Saturates, aromatics, resins and asphaltenes
GC	Gas chromatography
GC-MS	Gas chromatography–mass spectroscopy
HP-HT	High pressure–high temperature
NMR	Nuclear magnetic resonance
CHNS-O	Elemental analysis (Carbon, hydrogen, nitrogen, sulfur and oxygen)
FID	Flame ionization detector
TCD	Thermal conductivity detector
